# Fast intraoperative histology-based diagnosis of gliomas with third harmonic generation microscopy and deep learning

**DOI:** 10.1038/s41598-022-15423-z

**Published:** 2022-07-05

**Authors:** Max Blokker, Philip C. de Witt Hamer, Pieter Wesseling, Marie Louise Groot, Mitko Veta

**Affiliations:** 1 Department of Physics and Astronomy, Vrije Universiteit Amsterdam, Amsterdam, The Netherlands; 2 Department of Neurosurgery, Amsterdam UMC location VU University Medical Center, Amsterdam, The Netherlands; 3 Department of Pathology, Amsterdam UMC location VU University Medical Center, Amsterdam, The Netherlands; 4 Medical Image Analysis Group (IMAG/e), Department of Biomedical Engineering, Eindhoven University of Technology, Eindhoven, The Netherlands

**Keywords:** CNS cancer, Machine learning, Imaging techniques, Pathology

## Abstract

Management of gliomas requires an invasive treatment strategy, including extensive surgical resection. The objective of the neurosurgeon is to maximize tumor removal while preserving healthy brain tissue. However, the lack of a clear tumor boundary hampers the neurosurgeon’s ability to accurately detect and resect infiltrating tumor tissue. Nonlinear multiphoton microscopy, in particular higher harmonic generation, enables label-free imaging of excised brain tissue, revealing histological hallmarks within seconds. Here, we demonstrate a real-time deep learning-based pipeline for automated glioma image analysis, matching video-rate image acquisition. We used a custom noise detection scheme, and a fully-convolutional classification network, to achieve on average 79% binary accuracy, 0.77 AUC and 0.83 mean average precision compared to the consensus of three pathologists, on a preliminary dataset. We conclude that the combination of real-time imaging and image analysis shows great potential for intraoperative assessment of brain tissue during tumor surgery.

## Introduction

Gliomas represent approximately 80% of all malignant primary central nervous system (CNS) tumors diagnosed in the Western world^1^. Especially diffuse gliomas, characterized by progressive, infiltrative growth, have an immense impact on the patient’s life expectancy^2^. The World Health Organization (WHO) classification categorizes gliomas from low- to high-grade (I to IV) based on histological diagnosis and genotype^3^.

Management of low- and high-grade glioma patients relies on continuous advancements in the fields of tumor biology, imaging and treatment. In general, diffuse gliomas are incurable, and optimal treatment requires an invasive treatment strategy encompassing a combination of surgical resection, radiation therapy and chemotherapy. Extensive surgical resection is associated with delayed tumor progression for low-grade glioma patients, and improved patient survival for both low- and high-grade glioma patients^4^. The lack of a clear tumor boundary hampers the neurosurgeon’s ability to accurately detect and resect infiltrating tumor tissue. Current resection planning standards depend on T1-weighted gadolinium-enhanced or T2/FLAIR-weighted magnetic resonance (MR) imaging. In addition, more advanced pre-operative imaging techniques are available, such as multivoxel MR spectroscopy and positron emission tomography (PET)^5^. However, these techniques are unable to compensate for surgery-induced changes, like brain shift, and lack the ability to detect glioma infiltration on the cellular level^6^. The availability of non-invasive and label-free intraoperative imaging could enable neurosurgeons to visualize presence or absence of tumor during the resection procedure^7^.

We have shown before that third harmonic generation (THG) microscopy is capable of qualitative and quantitative visualization of glioma infiltration in ex-vivo brain tissue^8,9^. Our compact, portable multiphoton setup can be placed in the operating theatre and allows for label-free, real-time imaging of freshly resected tissue^10^. THG microscopy requires no tissue preparation and staining, in contrast to fluorescence and classical histological staining^11,12^. Additionally, in comparison to intraoperative MR imaging, THG provides high-resolution real-time imaging at the cellular level. Ultimately, it has the potential for in-situ assessment of brain tissue relying on endoscopic tools^8,13–15^.

Matching the rapid THG acquisition speed with real-time image analysis poses a challenge. Computer-assistance of the neurosurgeon during a surgical procedure should result in instant, meaningful histological feedback of the tissue state. Artificial intelligence (AI) is capable of delivering state-of-the-art computer assisted diagnosis^16–18^. Deep learning, a subfield of AI, allows for complex pattern recognition by means of an iterative, supervised optimization process. Compared to more classical machine learning algorithms, deep learning requires no manual feature extraction^19^. Given the substantial clinical and biological heterogeneity within gliomas, deep learning lends itself as a perfect extension for the detection and delineation of glioma infiltration with our THG setup.

Similar research in this field is based on topical application of fluorescent cell labeling agents, like moxifloxacin^20^, which inherently induces a certain waiting period before imaging takes place. Hyperspectral imaging (HSI) has also been applied in conjunction with deep learning to identify the boundaries of glioblastomas^21^, but entails a much lower resolution (128.7 $\upmu$m pixel size vs. 0.2 $\upmu$m for THG). The same principle applies to MR imaging^18^, aside from the impractical setup, high costs and time-intensive workflow. Hollon et al.^22^ combined stimulated Raman histology (SRH) with deep learning to demonstrate the potential of label-free microscopy in conjunction with AI. They utilized 2.5 million labeled patches from 415 patients, classified into 13 histologic categories, evaluated in a two-arm, prospective, multi-center, non-inferiority clinical trial. Ultimately, deep learning-based diagnosis of SRH images yielded 94.6% accuracy vs. 93.9% to pathologist-based interpretation of conventional hematoxylin and eosin-stained slides. Similar to our study, images obtained from samples inherited the patient-level diagnosis. Their results provide the foundation for our research, given the similar setup and goals. However, the SRH imaging speed is 8-fold slower (time to acquire 1 $\times$ 1 mm$^2$ is 120 s vs. 15 s for THG) compared to our portable THG system under identical resolution conditions^10^, and requires a more expensive and complicated setup.

In this study, we demonstrate the importance and effectiveness of pairing THG microscopy and deep learning in histology-based glioma vs. non-tumor diagnosis. We underline the value of removing noisy image data from the training set based on descriptive statistics. Building upon a dataset of 45 epilepsy (histologically normal brain tissue) and glioma tissue samples, from a total of 23 patients, we report 0.77 mean area under curve (AUC), 79% average binary accuracy, 60% sensitivity and 96% specificity. These results provide a proof-of-principle baseline for future research and require further evaluation on prospectively collected data. Ultimately, the work presented here serves as one more step towards equipping the neurosurgeon with the optimal tools to ultimately improve the outcome for patients with glioma.

**Figure 1:**
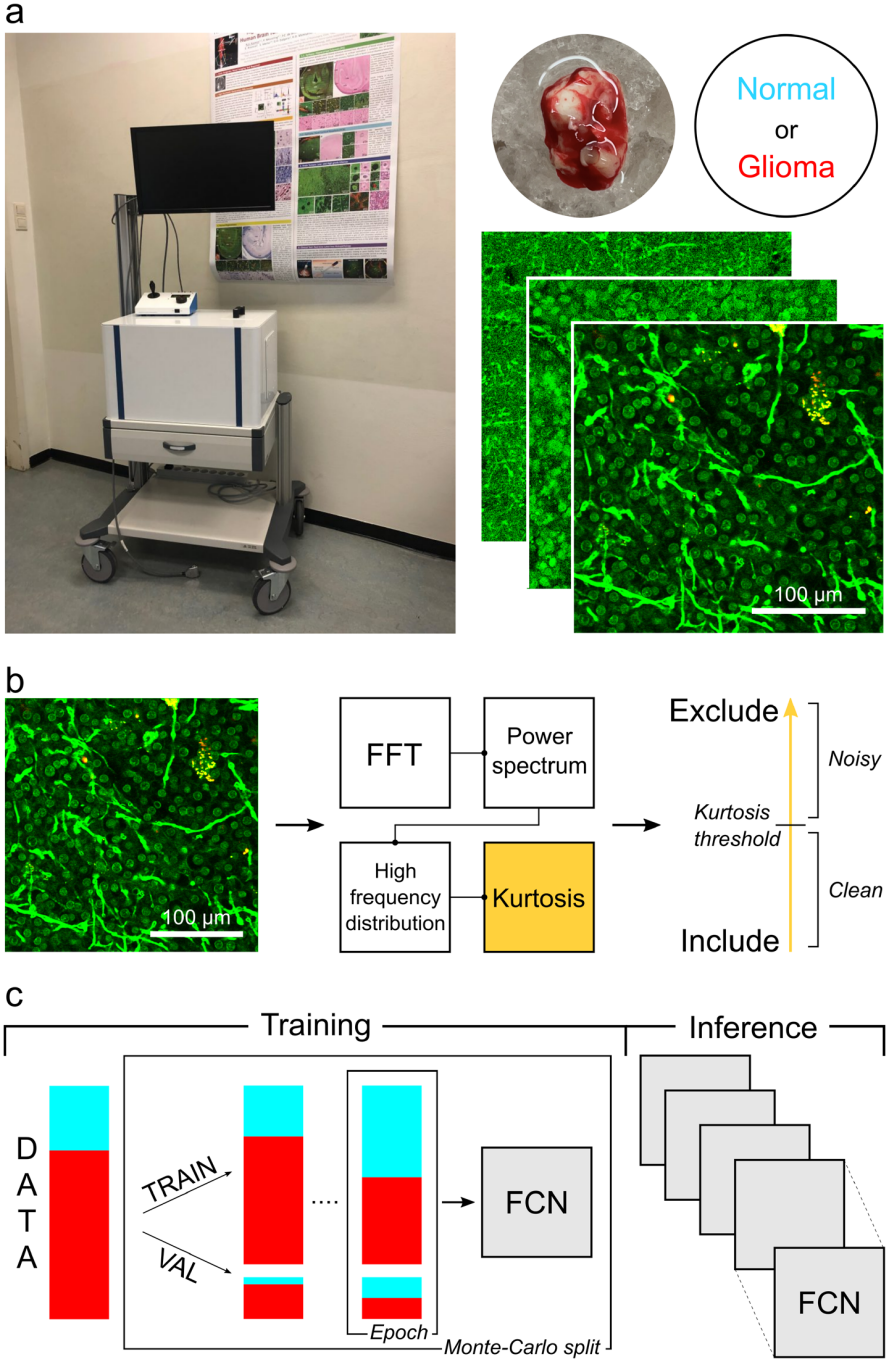
Complete tissue-to-inference pipeline with portable THG setup and deep learning. (**a**) Data acquisition using the portable THG setup (Flash Pathology B.V.). THG microscopy enables the acquisition of a ‘shadow contrast’ image, where myelinated fibers appear as bright curves and neuronal cell nuclei are dimly visible, while neuronal cell bodies appear as dark holes. Time to acquire image is approximately 3.5 s. (**b**) Data denoising by computation of descriptive statistics. Each image in the training set is processed to obtain the high frequencies distribution of the image’s frequency domain. Depending on the frequency distribution kurtosis, the image is either included or excluded from the training set. (**c**) Deep learning model training and inference on THG data, using Monte-Carlo cross-validation. The result is a series of optimized fully-convolutional models that are used during inference. The models are evaluated on a hold-out testing set.

## Results

In total 8 histologically normal brain samples from 5 epilepsy cases, and 37 tumor brain samples from 18 glioma cases were obtained from the neurosurgeon. With our THG microscopy setups, image data was acquired by loading and directly imaging of the fresh specimen. A training and testing set were constructed, as shown in Table 1. A complete list of included cases and specific patient diagnoses can be found as Supplementary Table S1. The images in the training set inherited the patient’s diagnosis (weak labels), while the images in the independent hold-out testing set were classified on the image-level by the consensus of 3 pathologists (strong labels). Image quality was assessed based on the high frequencies distribution in the image’s frequency domain power spectrum. Consequently, noisy image data was removed from the training set. Fully-convolutional networks (FCN) were optimized on the remaining training data, using Monte-Carlo cross-validation, and inference took place with the trained models on the testing set. During training, the imbalanced training and validation sets were balanced uniquely upon each epoch with data augmentation. An overview of the study’s tissue-to-inference pipeline is presented in Fig. 1.

**Table 1:** Brain tissue datasets for deep learning.

Dataset	# Epilepsy	# Glioma	# 2D images	Binary annotation
Training set	4	15	12,500	Image-level, inherited from diagnosis
Testing set	1	3	124	Image-level, consensus pathologists

**Figure 2:**
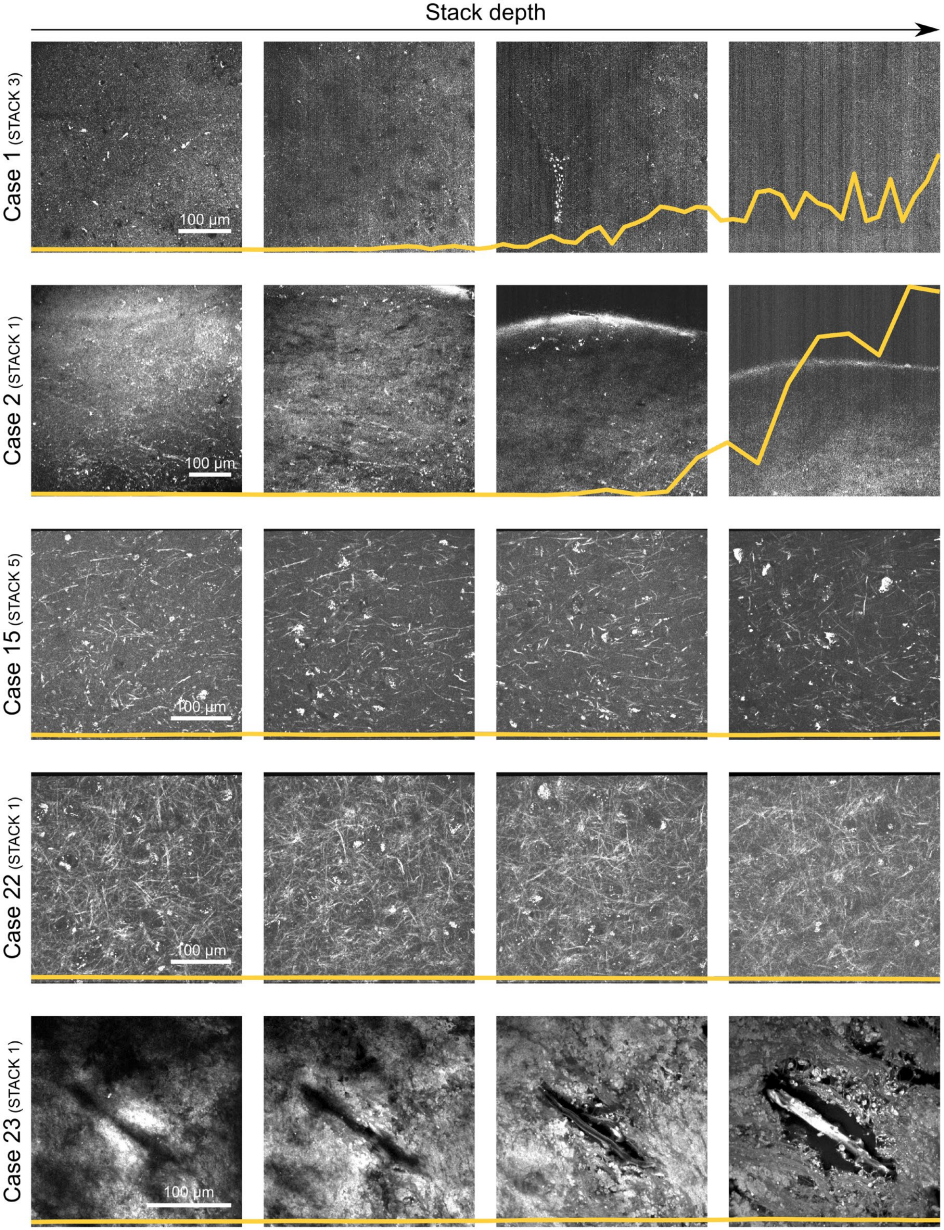
Montage of slices from the representative training subset for quality assessment. Each row showcases the first, two intermediate and last slice of the stacks picked from the subset cases. Each z-stack covers different structures over different imaging depths. The images highlight the quality heterogeneity across the subset: the slices from the first two cases show degrading quality in depth due to signal loss, while other stacks showcase no significant change. Each montage is overlaid with the stack’s kurtosis plot in yellow, visualizing kurtosis calculated across stack depth. The kurtosis was calculated on the top 20% frequencies of the power spectra. The plots are normalized with respect to the kurtosis maximum of the entire subset. Increase of kurtosis is observed for image slices from case 1 and 2, with respective loss of signal and gain of noise.

After image acquisition, and ahead of optimizing the deep learning models, noisy images were identified and dismissed from the training data. The ability to detect noise in our image data was examined by computing and comparing multiple descriptive statistics. For a small training subset, a variety of metrics were computed on each image’s frequency domain. In Fig. 2, example slices from this subset are shown in a montage. We identified kurtosis to be an adequate indicator of noise. Shown in yellow, the kurtosis is computed across image depth stacks and observed to increase as image quality degrades.

The data quality assessment relied on the Fourier transform of the image data. Beforehand it was unknown which power spectrum frequency and kurtosis cutoff values fit the glioma dataset best. To investigate, various selection criteria were constructed as seen in Table 2. The frequency threshold denotes the percentage of the power spectrum tail included in the kurtosis computations. The kurtosis threshold defines the kurtosis value above which images are excluded from the training set.

**Table 2:** Training data conditions and selection criteria.

Condition	Frequency threshold	Kurtosis threshold	# 2D images excluded
1	None	None	0 (0%)
2	40%	10	617 (5%)
3	40%	5	800 (6%)
4	20%	10	465 (4%)
5	20%	5	3416 (27%)
6	10%	10	376 (3%)
7	10%	5	10,113 (81%)

The number of images excluded from the training set reveals the ‘strictness’ of the chosen cutoff values. In the case of condition 7, as much as 81% of the entire training set was eliminated. There is a tight interplay between the frequency inclusion and kurtosis cutoff. To discover how our FCN model responds to these various training conditions and which performs best, we generated 10 random training-validation splits for each data condition, derived from the original training set (see Table 1). A FCN model was trained on each split, and repeated for all the data conditions, resulting in 10 trained models per condition. For data quality assessment, we first review the average validation set outcomes. These outcomes are presented in Table 3, indicated by the condition’s frequency inclusion and kurtosis exclusion criteria. Binary accuracy and receiver operating characteristic curve (ROC) AUC were averaged over the 10 splits, obtained at the lowest validation loss for each individual split. Classification threshold 0.5 is used for binary accuracy. The best performing training condition, number 4, is listed in bold. For this particular condition, 4% of the training data was excluded based on image quality (see Table 2), excluding images with a kurtosis higher than 10, calculated on the top 20% of the images’ frequency spectra. All quality-based conditions outperform models trained on the raw dataset (condition 1), with the exception of condition 7 which has the most strict selection criteria.

**Table 3:** Averaged validation results across 10 random training-validation splits using Monte-Carlo cross-validation.

Condition	Binary accuracy (%)	AUC
1 (None–None)	64.1 ± 7.4	0.70 ± 0.08
2 (40%–10)	72.0 ± 18.5	0.80 ± 0.16
3 (40%–5)	76.3 ± 16.7	0.81 ± 0.16
**4 (20%–10)**	**83.6** ± **13.6**	**0.90** ± **0.09**
5 (20%–5)	67.9 ± 19.3	0.75 ± 0.20
6 (10%–10)	75.6 ± 11.7	0.81 ± 0.11
7 (10%–5)	59.9 ± 12.8	0.58 ± 0.23

Optimal training parameters are in bold.

**Figure 3:**
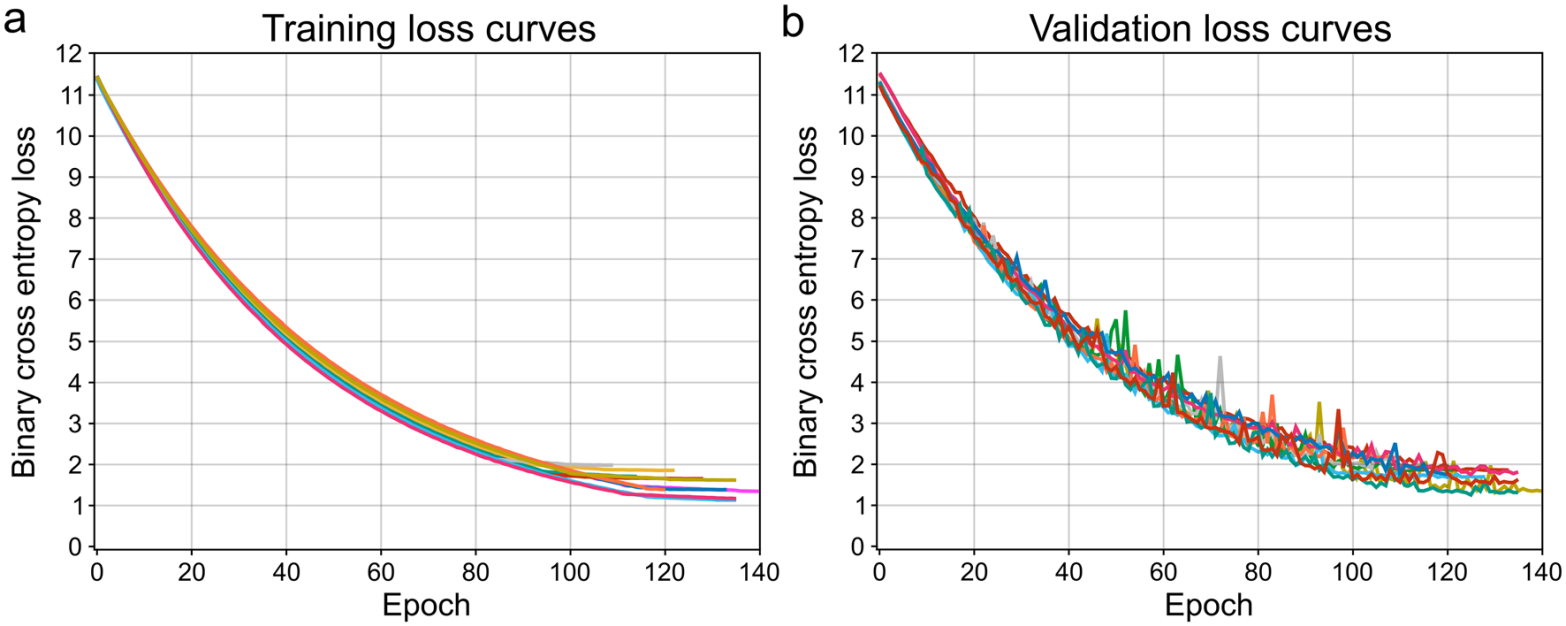
Loss curve plots of best performing training condition. (**a**) Training loss (binary cross entropy) is plotted against number of epochs for each model trained on the dataset of condition 4 (see Table 1) with Monte-Carlo cross-validation. (**b**) Validation loss (binary cross entropy) is plotted against number of epochs for each model trained on the dataset of condition 4 (see Table 1) with Monte-Carlo cross-validation. Both plots show decrease in loss over model training time, and start to converge around 120 epochs. Early stopping was employed when validation loss would not improve over 10 epochs. The loss function includes L2-regularization.

**Figure 4:**
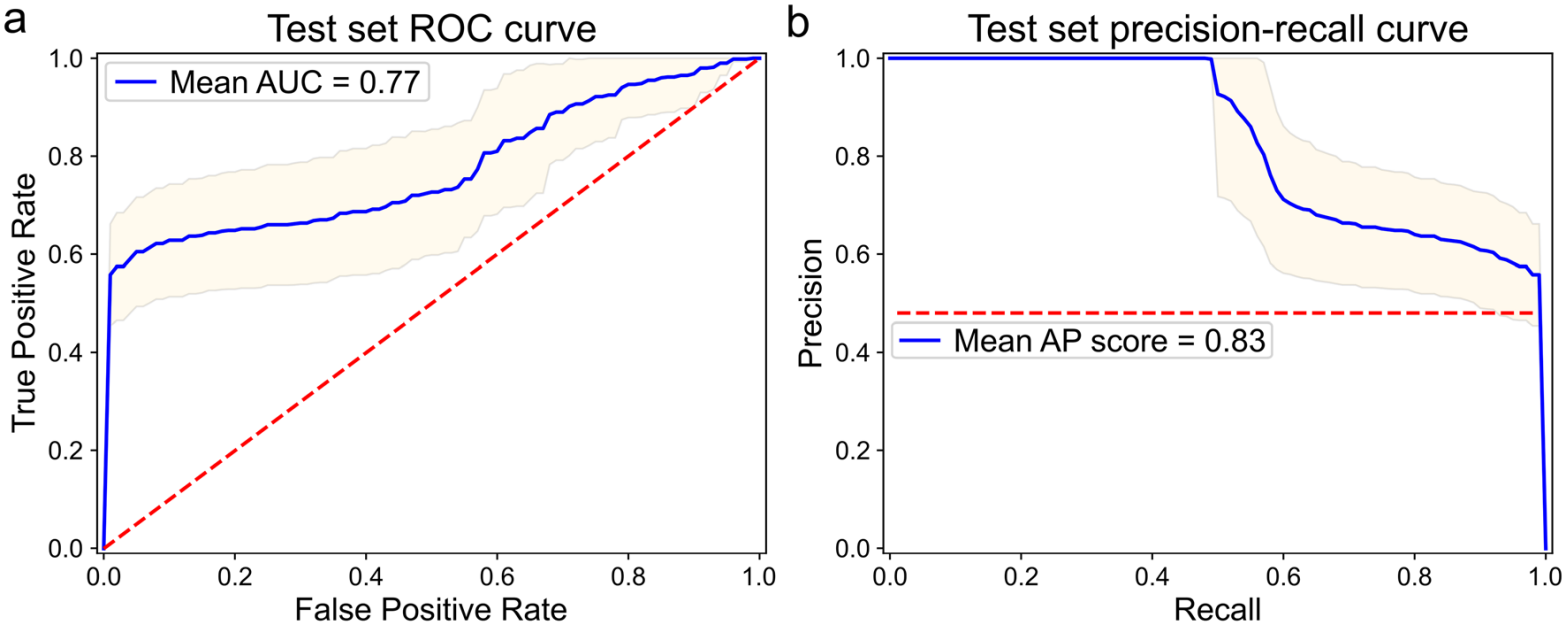
Interpretation of binary classification performance on hold-out test set. (**a**) Mean ROC curve (blue) and variance (yellow) of the 10 models from condition 4 (see Table 2) evaluated on the test set. For multiple thresholds, the true positive rate (TPR, also known as recall) is studied against the false positive rate (FPR). Dotted red line indicates a no-skill classifier. Mean AUC under ROC is reported. (**b**) Mean precision-recall curve (blue) and variance (yellow) of the 10 models from conditions 4 (see Table 2) evaluated on the test set. For multiple thresholds, the precision is studied against the recall (TPR). Dotted red line indicates a no-skill classifier. Mean average precision (AP) score is reported. The graphs explain performance with different metrics, and indicate no detriment in test performance due to imbalanced datasets.

The best performing condition identified which selection criteria were optimal for the training set. The models from this condition were then evaluated on the test set (see Table 1). No quality assessment took place on the test set itself. For each model, an ROC and precision-recall curve was produced, and we visualize the mean and variance of these in Fig. 4. We report a mean area under ROC curve of **0.77** ± **0.10** and mean average precision score of **0.83** ± **0.08**.

Calculating Youden’s J statistic on the individual ROC curves, the optimal classification threshold is determined for each model. From this, an average binary accuracy was obtained of **78.7 ± 5.6%** on the testing set. Finally, we report **60.3 ± 12.8%** sensitivity and **95.9 ± 4.2%** specificity.

**Figure 5:**
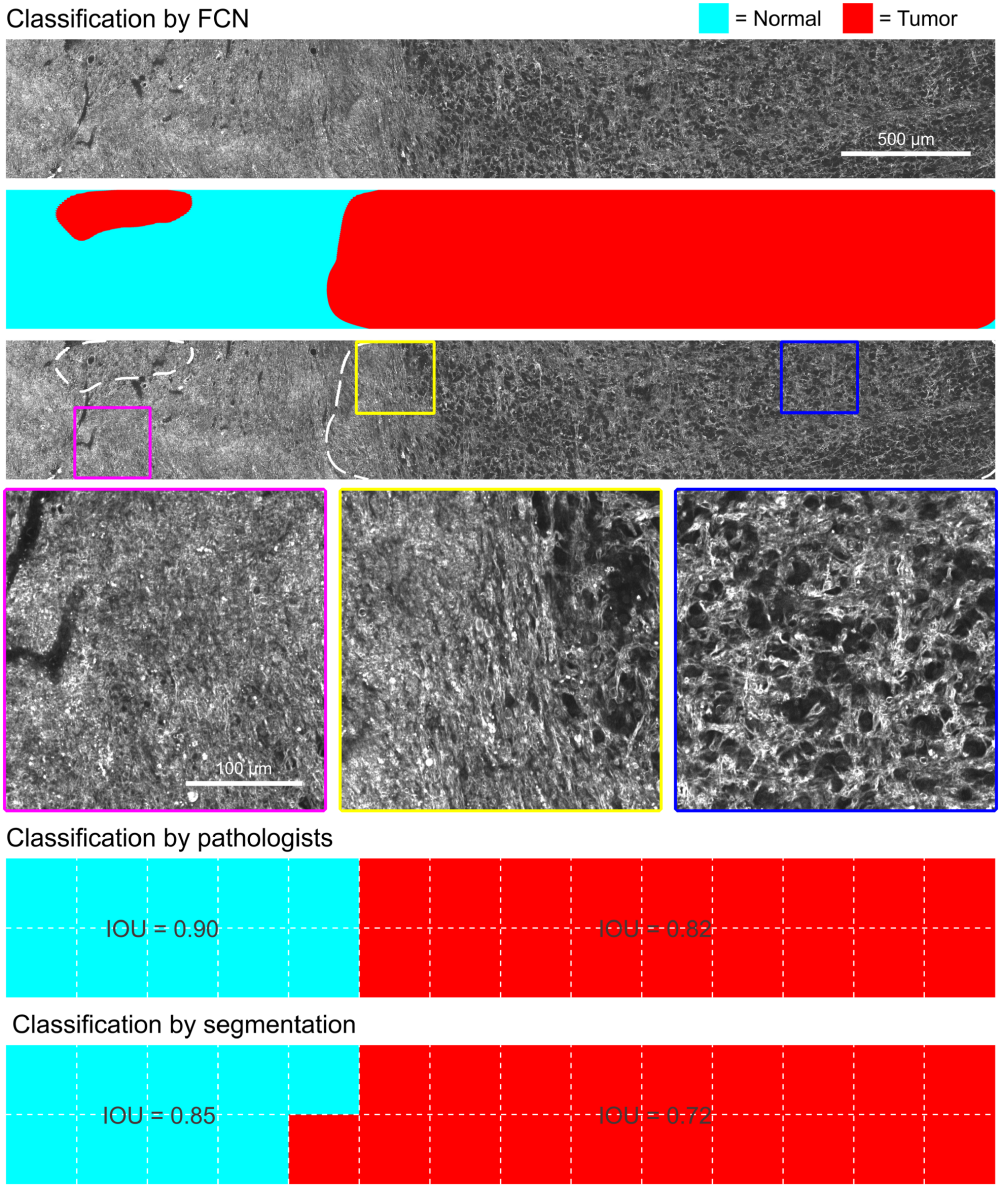
Semantic segmentation of low-grade glioma infiltration for test set case. Mosaic taken from test case 6 (see Supplementary Table S1). The mosaic shows clear gradually increasing density of tumor cells from histologically normal brain tissue (purple square) to tumor tissue (blue square). The tumor boundary (yellow square) is correctly identified by the FCN, as compared to the consensus of the pathologists.

The single 2D images in the test set are extracted from 4 mosaics (large tissue area scans) from 4 cases; 1 mosaic per case. Figure 5 visualizes classification performance of our top model on a test set case. The model picked performed best on the validation data from the best training condition (see Table 2). A visual comparison was made between the binary classification overlay from the FCN classification versus the segmentation algorithm from Zhang et al.^9^ and the consensus of the pathologists. The binary overlay was created by applying a sliding-window across each mosaic with stride 1. Each window is processed and classified by the model, and pixel scores are averaged over the number of classifications per pixel. This leads to the smooth binary overlay as shown the top-right corner of Fig. 5. The segmentation algorithm and pathologists overlays were taken from Zhang et al.^9^. The Jaccard index (intersection over union, IOU) between the semantic segmentation of the deep learning model and the reference overlays is computed per class. Compared to the consensus of the pathologists, we acquire **0.90** IOU for histologically normal image segmentation and **0.82** IOU for tumor segmentation. Compared to the segmentation map of Zhang et al.^9^, we obtain **0.85** normal IOU and **0.72** tumor IOU.

Finally, the FCN model is capable of very fast 2D image classification for achieving the above results. Single 2D images, 1000$\times$1000 pixels in size, were classified in **35 ms**.

## Discussion

The aim of this study was to demonstrate the importance and effectiveness of pairing THG microscopy and deep learning in glioma vs. non-tumor classification. The goal in mind is fast intraoperative pathology during tumor surgery to assist the neurosurgeon in optimal resection. Evaluation of the optimized models on the testing set revealed the generalization strength of our fully-convolutional networks, even when trained on a small and weakly labeled dataset, relying on patient diagnosis.

The image dataset consisted of a variety of field-of-views and tissue structure sizes. In addition, our acquired image dimensions vary due to the availability of multiple scan programs and developments to the experimental setup. Pixel size standardization was applied to each image to constrain network input to fixed sizes of brain tissue structures. Pseudo min-max normalization allowed for fixing data ranges and handling pixel outliers. To reinforce our algorithm for future use, the choice was made to design a fully-convolutional network, allowing dynamic input sizes, as opposed to a patch-based sampling algorithm^23^. This bypassed the need to decide an optimal patch size in which all the necessary diagnostic features are captured. The exact model architecture was determined based on trial-and-error on the validation set. Together with proper noise detection by kurtosis, a validation AUC as high as 0.90 was achieved after multiple iterations of model design (see Table 3). Further investigation of the optimal deep learning architecture for our dataset is necessary, and reserved for future work.

The dataset, which was largely inherited from our previous work^9^, consisted of an imbalanced training set and a balanced, independent testing set. Care was taken to avoid model bias towards the majority class by means of data augmentation and minority class oversampling during training. In addition, to avoid overfitting, data augmentation was applied, including random contrast, brightness and rotation adjustments. The pipeline feeding the FCN model was built in such a way that each epoch consisted of uniquely augmented and equally sampled images from both classes, during both the training and validation phase. The advantage of this pipeline is shown in Fig. 3 where training and validation loss curves are shown. The graphs indicate no signs of over- and underfitting of the models during Monte-Carlo cross-validation. While loss values are high, this is attributed to the use of L2-regularization during training, applied to all convolutional layer kernels, and specifically applied to combat overfitting.

During inference, ultimate test specificity turned out excellent (95.9% specificity) while test sensitivity lagged behind (60.3% sensitivity). In practice, this means our classifier is accurate in detecting tumor free cases, and less accurate in detecting tumor when it is present. One reason sensitivity is underperforming is the weak labelling of the glioma data. Epilepsy tissue, serving as a non-tumor indicator in lack of true healthy tissue, is strongly labelled as no acquired image will contain glioma tumor features. However, tissue obtained from glioma surgeries could consist of core tumor, peritumoral or normal appearing tissue features. Exact tissue sampling locations were not recorded. Therefore, image data acquired from glioma surgery samples were treated as bags of features, inheriting the patient-specific diagnosis. This data is therefore weakly labeled: tumor samples could include specimens with normal tissue characteristics. An approach could be employed where either the tissue sampling protocol is optimized, or multiple instance learning (MIL) is practiced^24^. Additionally, accurate annotation of healthy and tumor components in the image data can potentially improve the sensitivity^25^.

Our initial classifier performance is satisfactory, despite the data imbalance and weak positive class labels. This is visualized in Fig. 4a where the mean ROC curve and variance is plot for the test models. Each model is trained on a different random training-validation split, which explains how each model performs differently on the same, fixed test set. The variance could be an indicator of the overall heterogeneity of the training dataset. Since the ROC plot can be optimistic on imbalanced classification problems, Fig. 4b also displays the mean precision-recall curve and variance for the test models. A no-skill classifier is depicted in precision-recall as the number of positive examples in the dataset, in our case 60 out of 124 images (0.48). To summarize the graphs, we compute the mean area under ROC as 0.77 and precision-recall average precision as 0.83. Upon interpretation both the graphs and scores indicate similar performance when utilizing different metrics. This is expected as the test set is balanced, and minority oversampling took place during training.

An important step proved to be the identification and exclusion of noisy image samples based on kurtosis. The average binary accuracy and AUC on the validation set increased from 64% to 84% and from 0.70 to 0.90 respectively upon application of noise detection, leaving out only 4% from the entire training set. Kurtosis describes the shape of a probability distribution, being related to the tails of the distribution^26^. Calculated on the top frequencies of the images’ frequency power spectra, higher kurtosis relates to greater extremity of outliers in the distribution of these frequencies. This can be explained by the increase in variety and intensity of high frequencies in the Fourier domain due to addition of noise in the image domain^27^. The optimal frequency inclusion and kurtosis cutoff threshold were roughly determined using a representative subset as shown in Tables 2 and 3. Each training condition utilizing these thresholds outperformed the ‘raw’ training set, except for condition 7. This is explainable by the amount of training data excluded (81%) due to the tightly chosen parameters, leaving the network with little data to train on and therefore impacting performance. Finer-grained determination of these parameters could enable increased noise exclusion performance. Images with noise picked up by our approach were mainly the result of imaging depth limitations and subsequent signal loss. THG signal generation is limited by average laser power to avoid tissue damage, therefore limiting back-scattering beyond a certain penetration depth^28^. The maximum depth is generally not at issue when a human observer manually determines the imaging parameters before image acquisition. However, in anticipation of intraoperative application of this technology by an non-specialist end-user, automated noise detection and exclusion will remain of critical importance. The intra-operative, histology-based glioma diagnosis pipeline could benefit from increased custom noise exclusion in future iterations. In addition, image noise could be treated earlier in the tissue-to-classification pipeline. Deep learning methods exist to restore images without clean prior data using generative adversarial networks^29,30^. More importantly, at any stage of noise-induced image exclusion or cleaning, diagnostic ability of the classifier can be altered. It is extremely important no valid data is excluded or otherwise modified, during both training and inference of the algorithm. Additionally, out-of-distribution (OOD) detection could be equally important, where the classifier is able to detect out-of-bounds data^31^. More validation regarding noise detection and exclusion is necessary during the development of our classification pipeline.

The application of Monte-Carlo cross-validation to optimize 10 models independently, with randomly picked validation sets, largely ruled out the possibility of obtaining positive results by chance. One case from the negative class (epilepsy, histologically normal brain tissue) and 4 cases from the positive class (glioma, histologically tumor tissue) were randomly selected for validation, for each model. Given the heterogeneity and size of the dataset, a small set of cases with a variety of WHO grades and tissue sample locations, the generalization power of deep learning algorithms is underlined by our findings. Evidently, the inter-diagnostic differences between the histologically normal brain and glioma data classes are large and present enough in our data to allow for network convergence. While we have not focused on intra-diagnostic diagnosis, discriminating between low- and high-grade glioma for example, this may prove to be a much greater challenge if the dataset remains weakly labeled. However, inclusion of multiple histological classes^22^ in conjunction with image-level annotation could allow for this. The question is whether this kind of fine-grained decision making is necessary when aiming for tumor boundary detection.

Compared to earlier work from our research group on quantitative assessment of glioma presence, we report different results while employing deep learning classification. Zhang et al.^9^ implemented classification by segmentation based on active contour, performing quantitative analysis on the segmented neural cell bodies, nuclei and myelinated fibers. Evaluated on the same hold-out testing set (inherited from Zhang et al.), classification by segmentation reports an accuracy of 93%, compared to 79% from classification by deep learning. The scores reported here are measured against the agreement of 3 pathologists on the testing set. On average, the agreement between either 2 pathologists was 84%. The main difference between the outcome of these two approaches is the training dataset and its labeling. Zhang et al. utilized a small data subset (around 850 THG images), classified on the image-level by a trained observer. Training a convolutional neural network on such a small dataset is unfeasible, hence we opted for the inclusion of all available image data, even though the data is weakly labeled. Inclusion of more and diverse data allows for inherent regularization and generalization of the classification algorithm. However, a main advantage of deep learning classification is the inference speed in the order of milliseconds, compared to the much slower active contour segmentation speeds (in the order of minutes). This enables the possibility for instant feedback on our real-time acquired data. Our study provides a baseline which approximates the average agreement between either 2 pathologists who scored the testing set. While the approach would benefit from more accurate data, we demonstrate an effective classifier which is trained on imbalanced and weakly labeled data. Any future improvements in the availability of data and/or technical setup will therefore contribute to performance possibly the same or exceeding that of Zhang et al. Compared to classification by segmentation using active contour, we outperform in classification speed and allow inclusion of any available data, instead of only segmentable data.

Part of the steps towards clinical implementation of our classification pipeline in the portable setup, for on-site intraoperative pathology, is the required expansion of the dataset. Only a fraction of the future potential has been revealed here, with studies like Hollon et al.^22^ including over 400 patients. Enlarging the datasets, together with accurate recording of biopt sampling locations, will greatly advance our work on tumor tissue detection. Instead of implementing binary classification, a multi-class approach should be pursued in which a set of categories are learnt, ranging from definitive tumor free to definitely tumor. Uncertainty estimation and out-of-bound detection should play a pivotal role, moving our focus towards probable tumor infiltration detection^32^. This is the ultimate goal: enabling the neurosurgeon to intraoperatively probe tissue and receive a certainty estimation of presence versus absence of tumor. Is the piece of tissue that is being examined completely tumor free, and if not, how certain are we about the presence of tumor here? If the tissue of interest is not representative enough for accurate assessment, this should be detected and communicated back to the end-user as well.

The ultimate clinical implementation can be two-fold: ex-vivo analysis on resected tissue, and in-vivo endoscopic examination of tissue of interest^8,13^. The choice of implementation impacts the required classification pipeline. Image data from an endoscope might be of lower image quality and prone to movement artefacts, requiring proper pre-processing techniques before image classification. On the other hand, data from an ex-vivo tabletop device might cover tissue areas up to 1$\times$1 cm$^2$ and require parallelized sliding-window sampling algorithms. In addition, the choice of implementation impacts the strategy of data collection, as deep learning algorithms are sensitive to changes in acquisition devices and settings. Adversarial learning might prove useful here. In both implementations, a highly specific classifier like ours would be beneficial for the detection of true negative cases. It is extremely important no healthy tissue is resected during surgery, especially in the brain. Sensitivity is an aspect we will improve on in with the earlier mentioned enhancements of data acquisition.

In conclusion, we have shown a proof-of-principle to make meaningful impact in intraoperative, histologically-based glioma diagnosis by combining nonlinear optics with deep learning. Both of these techniques have seen major advancements over the last few years, and serve to complement each other. The combination of real-time histological imaging with with instant, deep-learning-based interpretation of the data has great clinical potential. Our classifier achieves on average 79% binary accuracy, 60% sensitivity and 96% specificity when compared to the consensus of 3 pathologists (with 84% average agreement between either 2 pathologists). The classifier enables instant histological feedback (35 ms) compared to classical classification by segmentation with active contour^9^, while allowing full inclusion of our microscopy dataset, with automated noise detection and exclusion. As the results are based on retrospective analysis, the classification pipeline would benefit from further, prospective data acquisition and validation of the deep learning model. Our focus will be the expansion of the dataset, model validation, and advancing towards the clinical implementation. Ultimately, the main goal is assisting the neurosurgeon in positively impacting glioma patient outcome.

## Methods

### Third harmonic generation microscopy

Higher harmonic generation is a nonlinear scattering process resulting from femtosecond laser light interaction with tissue material. Signal generation may occur in tissue depending on phase matching conditions and nonlinear susceptibility coefficients. The third order harmonic process results from the combination of 3 photons from the incident laser beam into 1 photon with triple the energy and thus one-third of the excitation wavelength, via a virtual energy state. Utilizing a laser wavelength in the near-infrared spectrum results in generation of the harmonic signal in the visible light range. The intensity of THG production is given by:

1 $$\begin{aligned} I_{THG}=\left( \frac{3\omega }{2n_{\omega }c}\right) ^2 \chi ^{(3)} I^3_{\omega } \int \limits ^{z_2}_{z_1}\frac{e^{i\Delta kz}}{(1+2iz/b)^2}dz, \end{aligned}$$

with laser intensity $I_{\omega }$, angular frequency $\omega$, medium refractive index $n_{\omega }$, speed of light *c*, phase mismatch $\Delta k=\left( n_{3\omega }3\omega /c\right) - 3\left( n_{\omega }\omega /c\right)$, position along the beam axis *z*, boundaries of the medium $z_1$ and $z_2$, and the confocal parameter of the focused laser beam *b*. Optimal THG production happens when a small inhomogeneity is introduced in the laser focus, or at structural interfaces. This makes THG microscopy an excellent candidate for brain tissue imaging, revealing neuronal cell bodies and their nuclei, and lipid-rich myeline.

### Human brain samples

Human brain tissue samples were acquired from 23 patients undergoing epilepsy surgery (5 cases) or glioma surgery (18 cases) in the Amsterdam University Medical Center (Amsterdam UMC). All patients gave a written informed consent for tissue biopsy collection and signed a declaration permitting the use of their biopsy specimens in scientific research. The research was conducted in accordance with the Netherlands Code of Conduct for Research Integrity and the Declaration of Helsinki, and the protocol was approved by the Medical Ethical Committee of the Amsterdam UMC (W18_354 # 18.402). A list of the histological diagnoses can be found as Supplementary Table S1.

During brain surgery, small pieces of tissue (1.0$\times$1.0$\times$0.5 cm) were excised and placed in a sample holder ($\mu$-Dish 35 mm, high, Ibidi GmbH), rinsed with artificial cerebrospinal fluid (aCSF), cut with a surgical scalpel to generate flat surfaces, and imaged. The majority of the included patient samples were inherited from Zhang et al.^9^ and imaged on the lab setup. Over time, a portable setup (Flash Pathology B.V.) was acquired and situated in the hospital to allow for close collaboration with the neurosurgery department and advance research in other fields of interest^10^. This serves as a stepping stone towards utilizing the portable device to assist the neurosurgeon with tumor boundary recognition on freshly resected tissue, during surgery. Accordingly, the most recent acquired patient samples moved from lab image acquisition to portable imaging in the interest of evaluating the portable microscopy device. The histological diagnosis of all cases was based on analysis by the pathology department.

### Microscopy setups

Imaging of the acquired brain tissue samples was performed on one of two setups: the lab setup or portable setup. While the setups are different, the intrinsic signal generation is the same, and both systems should therefore reveal the same histological hallmarks in the brain samples with similar quality.

The lab setup utilizes an optical parametric oscillator (Mira-OPO, APE GmbH) pumped at 810 nm by a Ti:Sapphire laser (Chameleon Ultra II, Coherent Inc.), generating 200 fs pulses at 1200 nm and a repetition rate of 80 MHz. The beam from the OPO was delivered into a commercial two-photon laser-scanning microscope (TriM Scope I, LaVision BioTec GmbH) and focused on the sample using a water-dipping objective (25$\times$/1.10 NA, Nikon). The generated and back-scattered THG photons at 400 nm were split from the fundamental laser photons by a dichroic mirror (T800LPXRXT, Chroma Technology GmbH), filtered by a narrow-band interference filter (Z400/10X, Chroma Technology GmbH) and collected with a high sensitivity GaAsP photomultiplier tube (H7422-40, Hamamatsu Photonics K.K.).

The portable setup utilizes a cart with a multiphoton microscope (FD1070, Flash Pathology B.V.) employing 50 fs pulses at 1070 nm (Fidelity 2, Coherent Inc.). Nonlinear signals are collected in backscatter direction by the inverted oil-immersion objective (40$\times$/1.3 NA, Nikon) and separated into three detection arms. Using analogue photomultiplier tubes (H10720 and H7422, Hamamatsu Photonics K.K.) these three arms capture distinct multiphoton signals: third harmonic generation (THG) at 355/5 nm, second harmonic generation (SHG) at 535/5 nm and two-photon excited fluorescence (2PEF) at 562–665 nm. In line with the data acquired from the lab setup, only the generated THG signals are utilized in this project.

### Image acquisition

Images were acquired from the tissue samples utilizing acquisition software developed by Flash Pathology B.V. on both our setups. Regions of interest were identified in inspection mode, and imaged using several scanning modes. A variety of scanning modes allowed the creation of a diverse dataset with not only single-view 2D shots, but including structural depth information (3D depth scans) as well as large views of the tissue samples (2D mosaics of stitched tiles). Images are acquired at 0.29 Mpixel $s^{-1}$. Collected THG signals were saved in 16-bit TIFF files.

Afterwards, the samples were fixated and processed by the pathology department for H&E staining and one-to-one image comparison with the acquired data. The majority of the images inherited the patient diagnosis on the image level and together formed the training set for the classifier. Mosiacs generated from cases 6, 12 and 17 (see Supplementary Table S1) were surveyed independently by 3 pathologists at the image level, and therefore served as an independent testing set. One epilepsy case (case 9) was added to this set for reference purposes. All image data retrieved from tissue samples was summed to a number of 2D images per set.

The training set is heavily imbalanced (9.7% histologically normal vs. 90.1% glioma images), which is compensated for with minority class oversampling during training. The hold-out testing set is balanced (64 histologically normal vs. 60 glioma images). For structure size standardization, each image was resized to 0.5 $\upmu$m pixel size. Images were then pseudo min-max normalized, with the 0.01 and 0.99 percentiles respectively, and rescaled to 8-bit range. Table 1 lists the number of 2D images available to the model for training and evaluation.

### Data quality assessment

Inspection of the training set revealed prominent variations in image quality. These can be mainly attributed to increase in noise due to loss of signal along the imaging depth. A measure of data quality was implemented to identify and exclude these images, to avoid disruption of classifier performance.

Based on the insights of Koho et al.^27^, frequency domain analysis was performed on the training set. In order to detect noise, descriptive statistics for each image were computed from the image’s frequency power spectrum. An increase in noise in the image domain leads to an increase of variation and pitching in the higher frequencies in the frequency domain. This can be quantified with statistical metrics. In order to determine the best performing statistical measure for this specific use case, a representative subset of the training set was assessed. Z-stacks from two epilepsy, two low-grade and one glioblastoma cases were included in this subset.

A MATLAB workflow based on the methods of Koho et al.^27^ was built to produce power spectra for the subset image slices. A number of statistical metrics were computed on each slice’s frequency domain: mean, inverse mean, standard deviation, inverse standard deviation, coefficient of variation, kurtosis and skewness. Various portions of the spectrum tail were included to investigate optimal frequency inclusion.

Comparing the computed metrics with the visual image quality, we determined the optimal noise descriptor and constructed various training conditions for the binary classifier.

### Binary classifier training and evaluation

A fully-convolutional network (FCN) was built for optimization on the binary classification task. The model has a 6-block architecture, with an additional sigmoid classification layer. The first 4 blocks each consist of 5$\times$5 convolution, followed by batch normalization, rectified linear unit (ReLU) activation and 2$\times$2 max pooling. The convolutional layers in these blocks comprise of 32, 64, 128 and 256 neurons, respectively. The fifth block consists of 1$\times$1 convolution with 64 neurons, followed by batch normalization and ReLU activation. The sixth block concludes the model with 1$\times$1 convolution with 1 neuron and global average pooling. Each convolutional layer is L2-regularized with factor 0.01. Furthermore, each convolutional layer’s kernel weights are initialized with He uniform variance scaling.

Monte-Carlo cross-validation is applied to produce 10 random training-validation splits per training condition. One case from the negative class and 4 cases from the positive class were randomly selected from the training set. Both the training (used to find model parameters) and validation (used to find model hyper parameters) set were balanced by oversampling of the negative class during optimization of the model. Slices were processed, shuffled and uniquely augmented each iteration with random contrast, brightness and rotation adjustments. Pixel values were converted to floating points and clipped to [0, 1]. Given that the model is a fully-convolutional one, padded batches of 32 slices were generated and fed into the model.

Models were optimized using the SGD optimizer with 0.9 momentum and initial learning rate of 1e−4. The learning rate was reduced on each validation loss plateau with factor 0.2, and early stopping was employed. The validation binary accuracy and AUC at the lowest validation loss were recorded and averaged for each training condition and fold. Finally, all models of the best performing training condition were evaluated on the hold-out testing set. The test set average binary accuracy, area under ROC curve, average precision, sensitivity and specificity are reported, relying on Youden’s index for finding the optimal classification threshold.

The models were produced, trained and evaluated with TensorFlow 2.4.1^33^ on a 4-GPU workstation (Lambda Quad, Lambda Labs, Inc.).

## Supplementary Information

Supplementary Information.

## Data Availability

Image data and trained deep learning models are provided as part of the replication package. It is available at: https://dataverse.nl/dataset.xhtml?persistentId=doi:10.34894/G8PZKV.

## Code availability

Code for data processing and deep learning is provided as part of the replication package. It is available at: https://dataverse.nl/dataset.xhtml?persistentId=doi:10.34894/G8PZKV.
