# Supplementary material for: Fast intraoperative histology-based diagnosis of gliomas with third harmonic generation microscopy and deep learning
Source: Sci Rep. 2022 Jul 5;12:11334. doi: 10.1038/s41598-022-15423-z (PMC9256596; doi:10.1038/s41598-022-15423-z)
Supplement: Supplementary file 1 — Supplementary Information. [file 41598_2022_15423_MOESM1_ESM.pdf]

Fast intraoperative histology-based diagnosis of gliomas with third harmonic generation microscopy and deep learning

Max Blokker[1,*], Philip C. de Witt Hamer[2], Pieter Wesseling[3], Marie Louise Groot[1], and Mitko Veta[4]

[1]Department of Physics and Astronomy, Vrije Universiteit Amsterdam, Amsterdam, The Netherlands
[2]Department of Neurosurgery, Amsterdam UMC location VU University Medical Center, Amsterdam, The Netherlands
[3]Department of Pathology, Amsterdam UMC location VU University Medical Center, Amsterdam, The Netherlands
[4]Medical Image Analysis Group (IMAG/e), Department of Biomedical Engineering, Eindhoven University of Technology, Eindhoven, The Netherlands
[*]m.blokker@vu.nl

Supplementary information

Table S1. Histological diagnosis and origin of human brain tissue samples; summary between brackets: World Health Organization (WHO) grade of the gliomas based on histology, and where available IDH-status as assessed by immunohistochemistry for IDH1 R132H mutant protein; from patients undergoing epilepsy surgery (case 1, 9, 16, 18, 22) histologically normal brain tissue was used; for each case, the number of different tissue samples used for this study is given by the Arabic numeral at the end (for case 1-23 in total 45 samples).

Case	Histological diagnosis	# Samples
1	Non-neoplastic	2
2	Diffuse astrocytoma (II); left frontal lobe	2
3	Focally anaplastic diffuse glioma (III); right parietal lobe	1
4	Oligo-astrocytoma (II); left frontoparietal lobe	1
5	Oligo-astrocytoma (II); right parietal lobe	1
6	Oligodendroglioma (II); right insular region	1
7	Oligodendroglioma (II); right insular region	3
8	Diffuse glioma (II); left frontal lobe	3
9	Non-neoplastic	1
10	Oligodendroglioma (II); right temporo-insular region	3
11	Glioblastoma (IV)	4
12	Anaplastic oligodendroglioma (III; IDH-mutant); right parietal lobe	1
13	Glioblastoma (IV; IDH-wildtype); right temporal lobe	2
14	Astrocytoma (II; IDH-mutant); left frontal lobe	2
15	Glioblastoma (IV; IDH-mutant); right frontal lobe	2
16	Non-neoplastic	1
17	Astrocytoma (II; IDH-mutant); right frontal lobe	1
18	Non-neoplastic	2
19	Glioblastoma (IV; IDH-wildtype); left frontal lobe	3
20	Astrocytoma (II; IDH-mutant); left frontal lobe	2
21	Glioblastoma (IV; IDH-wildtype); right insular region	3
22	Non-neoplastic	2
23	Astrocytoma (II); left frontal lobe	2
**Total**		**45**
